# A survey of instructions to authors in surgical journals on reporting by CONSORT and PRISMA

**DOI:** 10.1308/003588412X13373405386619

**Published:** 2012-10

**Authors:** S Shantikumar, J Wigley, W Hameed, A Handa

**Affiliations:** ^1^Oxford University Hospitals NHS Trust,UK; ^2^Buckinghamshire Healthcare NHS Trust,UK

**Keywords:** Editorial policies, Clinical trials, Conflict of interests, Journalism

## Abstract

**INTRODUCTION:**

Guidance has been published on how best to report randomised controlled trials (Consolidated Standards of Reporting Trials – CONSORT) and systematic reviews (Preferred Reporting Items for Systematic Reviews and Meta-Analyses – PRISMA). The aim of this study was to establish to what extent surgical journals formally endorse CONSORT and PRISMA in the respective reporting of randomised controlled trials and systematic reviews.

**METHODS:**

Overall, 136 surgical journals indexed in *Journal Citation Reports®* were studied. Author guidelines were scrutinised for the following guidance: conflict of interests (COI), the Uniform Requirements for Manuscripts (URM), clinical trial registration, CONSORT and PRISMA.

**RESULTS:**

The frequency of guidance endorsement was found to be as follows: COI 82%, URM 62%, trial registration 32%, CONSORT 29% and PRISMA 10%. Journals with a higher impact were more likely to adopt trial registration, CONSORT and PRISMA. Journals with editorial offices in the UK were more likely to insist on disclosure of COI and to endorse CONSORT.

**CONCLUSIONS:**

Guidelines produced to improve publication practice have not been implemented widely by surgical journals. This may contribute to an overall poorer quality of published research. Editors of surgical journals should uniformly endorse reporting guidance and update their instructions to authors to reflect this.

In an age of burgeoning research in surgery, there is a need to improve the standards of reporting. Randomised controlled trials (RCTs) and systematic reviews offer the highest quality research and guidance has been well publicised as to how best to report these. First published in 1996^1^ and most recently updated in 2010,^2^ the Consolidated Standards of Reporting Trials (CONSORT) statement provides recommendations on how to report parallel group RCTs. A similar guideline exists for the reporting of systematic reviews: initially named QUOROM (Quality of Reporting of Meta-analyses),^3^ it was updated in 2009 and published under PRISMA (Preferred Reporting Items for Systematic Reviews and Meta-Analyses).^4^

Both CONSORT and PRISMA contain a checklist of items that are essential for transparent reporting. A recent systematic review revealed that journal adaptation of the CONSORT guidelines resulted in an improvement in reporting of RCTs.^5^ Furthermore, a survey of highest impact medical journals found that only a third required CONSORT in the reporting of RCTs.^6^ No such evidence exists yet for the implementation of PRISMA in the current literature.

All relevant information about a study should be reported to allow readers and users of the research to assess its validity. The editorial practice in surgery to this regard has not yet been scrutinised. The aim of this study was to establish to what extent surgical journals formally endorse CONSORT and PRISMA, as well as other published guidance, in the respective reporting of RCTs and systematic reviews.

## Methods

The science edition of the 2009 *Journal Citation Reports®* (Thomson Reuters, New York, US) was accessed via http://apps.isiknowledge.com/ on 8 March 2011. All journals listed in the subject category ‘surgery’ were identified. Journals that did not publish original research were excluded. The websites for included journals were accessed and the instructions to authors were downloaded in March 2011. Each document was read to establish endorsement of the CONSORT and PRISMA guidelines. The impact factor and geographical location of the journal was also noted, as were endorsements of: disclosure of conflict of interests (COI), trials registration and the Uniform Requirements for Manuscripts Submitted to Biomedical Journals (URM).^7^ The geographical locations of editorial offices were subdivided into the UK, Europe (without the UK), North America (US and Canada) and other countries.

For each of the guidelines and endorsements noted above, we recorded whether it was mandatory (ie required for acceptance of the article), recommended or not specified. Data were collected independently by two authors (SS, JW). Discrepancies occurred in 3% of recorded items. These were resolved by mutual discussion along with a third author (WH) with reference to the original documents.

Impact factor^8^ was analysed as a continuous variable and compared with reporting frequency using logistic regression. Odds ratios are reported with a 95% confidence interval for each 1-unit change in impact factor. The associations between geographical region and the presence of journal recommendations were calculated using Pearson’s chi-squared test for proportions, with subgroup analyses calculated using the post-hoc** Marascuilo procedure. As all chi-squared tests were performed with four groups, these were analysed with three degrees of freedom. All statistical analyses were performed using SPSS® v18 (SPSS, Chicago, IL, US).

## Results

We identified 167 journals in the *Journal Citation Reports®* category ‘surgery’. Of these, 31 were excluded for the following reasons: journal not in English (*n*=20), no original research published (*n*=5), not a surgery journal (*n*=4: 2 pathology, 1 anaesthesia, 1 animal surgery) and author guidelines not found on website (*n*=2). Overall, 136 journals were studied with an impact factor ranging between 0.1 and 7.9.

The distribution of impact factors is shown in Table 1. Most surgical journals had an impact factor below 2. The geographical distribution of editorial offices can be seen in Table 2, with the majority of journals based in North America.

**Table 1:** Association between journal impact factor and endorsement of reporting guidelines

	Impact factor				OR (95% CI)	*p*-value
	**0–1** (*n*=45, 33%)	**1–2** (*n*=44, 32%)	**2–3** (*n*=29, 21%)	**>3** (*n*=18, 13%)		
URM	25 (56%)	25 (57%)	20 (69%)	14 (78%)	1.2 (0.9–1.7)	0.14
COI	33 (73%)	37 (84%)	24 (83%)	17 (94%)	1.4 (0.9–2.1)	0.10
Trial registration	7 (16%)	14 (32%)	9 (31%)	12 (67%)	1.8 (1.3–2.5)	**<0.001**
CONSORT	5 (11%)	14 (32%)	9 (31%)	12 (67%)	1.8 (1.3–2.5)	**<0.001**
PRISMA	0 (0%)	3 (7%)	5 (17%)	5 (28%)	1.7 (1.2–2.5)	**0.004**

URM = Uniform Requirements for Manuscripts; COI = conflict of interests; CONSORT = Consolidated Standards of Reporting Trials; PRISMA = Preferred Reporting Items for Systematic Reviews and Meta-Analyses; OR = odds ratio; CI = confidence interval

**Table 2:** Association between geographical location of journal and endorsement of reporting guidelines

	North America (*n*=74, 54%)	Europe (except UK) (*n*=38, 28%)	UK (*n*=13, 10%)	Other (*n*=11, 8%)	*p*-value
URM	50 (68%)	17 (45%)	10 (77%)	7 (64%)	0.07
COI	61 (82%)	29 (76%)	13 (100%)	8 (73%)	0.24
Trial registration	26 (35%)	7 (18%)	7 (54%)	4 (36%)	0.09
CONSORT	25 (34%)	4 (11%)	9 (69%)	2 (18%)	**<0.001**
PRISMA	11 (15%)	1 (3%)	1 (8%)	0 (0%)	0.12

URM = Uniform Requirements for Manuscripts; COI = conflict of interests; CONSORT = Consolidated Standards of Reporting Trials; PRISMA = Preferred Reporting Items for Systematic Reviews and Meta-Analyses

The URM were mentioned in 84 journals (62%). Of these, 57 gave a web reference, 9 a print reference, 7 both a web and print reference, and 11 no reference at all. Of the 18 journals that provided a print reference to URM, only 1 was in date. There was no association between impact factor and reference to URM (Table 1), neither was there a difference in the reporting frequencies in different locations (Table 2).

A statement on COI was given in 111 journals (82%). There was no relationship between the requirement of a COI statement and impact factor although UK-based journals were more likely to insist on this than North American and other European journals (*p*<0.01 for both).

Clinical trial registration was mentioned in 44 journals (32%). It was compulsory for consideration of publication in all but three of these. Twenty-three of these journals gave examples of relevant clinical trial registry sites. Journals with a higher impact were more likely to require trial registration (*p*<0.001).

Endorsement of the CONSORT guidelines was found in only 40 journals (29%). Of these, it was compulsory for consideration of publication of RCTs in 36. In the remainder, it was either recommended (*n*=2) or unclear (*n*=2). In these 40 journals, 33 gave a website reference, 4 a print reference, and 1 both a print and web reference. Two journals did not provide any form of reference. Of the five print references, none were in date. CONSORT was most likely to be required by higher impact journals (*p*<0.001). UK journals asked for CONSORT significantly more frequently than the non-North American journals (*p*<0.01 for all).

Guidelines for systematic reviews (either QUOROM or PRISMA) were endorsed by only 13 journals (10%). Of these, nine required these for submission. Ten journals gave a web reference and three a print reference. None gave both. Of the three print references, only one referenced the recent PRISMA guidelines. The other two referenced the outdated QUOROM statement. Systematic review guidelines were also more likely to be asked of by higher impact journals (*p*=0.004). This was not biased towards a particular geographical location.

## Discussion

In this study, the instructions for authors of indexed surgical journals were surveyed to see if improvements in practice could be made. The general endorsement and application of published guidelines was found to be low although journals with a higher impact factor did tend to implement these more frequently.

A 2008 study by Hopewell *et al* looked at author instructions of the top five high impact journals across the medical specialties.^6^ Of the 165 journals studied, 37% endorsed trial registration and 38% endorsed CONSORT. These figures are slightly higher than ours. However, as they only looked at the top impact journals, their sample is likely to be skewed. Schringer *et al* also looked at the methodological content of author instructions in high profile medical journals.^9^ They also found that very few endorsed CONSORT (22%). The poorer endorsement seen here, considering the authors looked at high impact medical journals, is likely to be a reflection of the fact that this study was conducted some years ago (2006).

A similar study from 2010 looking at editorial policies in 69 indexed paediatric journals by Meerpohl *et al* found similar deficiencies, with 23% mentioning trial registration and 20% endorsing CONSORT.^10^ Although these figures are lower than those found for surgical journals in the present study, the results are of a similar order. Matarese contrasted the author guidelines between UK and Italian journals, and found that the Italian journals scored comparatively poorly in their editorial policies.^11^ In line with this, our study found that the UK surgical journals more consistently endorsed recognised guidelines than those from elsewhere in Europe.

Reporting guidelines such as CONSORT and PRISMA have been developed collaboratively to help improve the quality of research reports. Evidence exists to demonstrate their positive impact on reporting.^5,12^ Our study demonstrates that CONSORT is used by 29% of surgical journals. However, those that referred to a print version used out-of-date references and it would be prudent for journal editors to keep their author guidelines updated. None of the above studies reported on the endorsement of PRISMA specifically. We found that PRISMA was the most rarely adopted of the studied guidelines, with only 10% of surgical journals insisting on or suggesting its use. This issue needs addressing urgently given the rapid increase in the rate of publication of systematic reviews.^13^

Our study does have limitations. First, not all surgical journals are indexed in the *Journal Citation Reports®* so we may not have studied a representative sample. However, given that we found that lower impact journals less frequently endorse reporting guidelines and that higher impact journals tend to be indexed, the true frequency of endorsement may be even lower. We did not survey journal editors themselves; it may be that editorial offices have implicit policies that are not reflected in their guidelines. However, if this is the case, we suggest that journals update their guidelines in light of this. Indeed, published instructions to authors would benefit from being dated and revised regularly to avoid overlooking updated reporting guidance. As it stands, it is unclear if this generally occurs with regularity. Finally, we used impact factor as a surrogate for journal profile despite its known weaknesses.^14^

## Conclusions

We found that guidelines produced to improve publication practice have not widely been implemented by surgical journals. Not all surgical papers are published in surgical journals and many high quality reports are found in the more general medical literature. In addition, just because certain journals do not insist on good quality reporting, we are not suggesting that the majority of articles published therein are of inadequate quality. However, there is evidence to suggest that certain articles in the field are not conducted and reported appropriately.^15–17^

Good publication practice is vital for two reasons: to ensure the publication of good quality trials and reviews, and to allow the users of the research to assess its validity. While some of this responsibility certainly rests with authors, standards could be improved if journal editors insisted on good quality reporting. We feel that this is an appropriate time to suggest that surgical journal editors uniformly endorse such guidance and update their instructions to authors.
